# Composition Study of Polyphyllin in *Paris polyphylla* by Ultrasound-Assisted Deep Eutectic Solvent Extraction Combined with UHPLC-MS/MS

**DOI:** 10.3390/molecules31030473

**Published:** 2026-01-29

**Authors:** Jinyu Guo, Jiajia Liu, Minlong Li, Zhenlin Tan, Huayin Lu, Yuting Zhou

**Affiliations:** School of Pharmacy, Youjiang Medical University for Nationalities, Baise 533000, Chinaluhuayin11@163.com (H.L.); z3527508073@163.com (Y.Z.)

**Keywords:** polyphyllin I, *Paris polyphylla*, ultrasound-assisted deep eutectic solvents, UHPLC-MS/MS, orthogonal design

## Abstract

*Paris polyphylla* (Chonglou), a medicinal herb documented in Shennong’s Classic of Materia Medica and a key component of formulas such as Yunnan Baiyao, is a rare and endangered plant prized for its bioactive steroidal saponins, notably polyphyllin I (PPI) and II (PPII). However, its pharmacological potential is hampered by inefficient extraction and unreliable compound identification. Herein, we developed a sustainable and efficient extraction strategy using ultrasound-assisted deep eutectic solvents (DES), optimized via an L_9_(3^4^) orthogonal experimental design. Extraction efficiencies across the seven *Paris* species ranged from 2.04% to 16.51%, achieved by systematically optimizing key parameters such as the choline chloride-to-ethanol molar ratio (1:1.8), material-to-liquid ratio (1:20 g mL^−1^), and extraction time (100 min). By ultra-high-performance liquid chromatography–tandem mass spectrometry (UHPLC-MS/MS) analysis, PPI and PPII were quantified using specific retention times and characteristic fragment ions, revealing content ranges of 3.282–21.452 mg g^−1^ and 4.201–17.975 mg g^−1^, respectively. This methodology provides a robust platform for quality control and standardization of *Paris*-derived medicines, while paving the way for sustainable utilization and in-depth study of its steroidal saponins.

## 1. Introduction

Traditional Chinese medicine (TCM) has long harnessed the therapeutic potential of medicinal plants, with *Paris polyphylla* (commonly known as Chonglou) standing as a cornerstone in this ancient practice [1]. Recognized for centuries in classical texts such as *Shen Nong Ben Cao Jing*, this perennial herb from the Liliaceae family is primarily distributed in regions like Yunnan, Guizhou, and Sichuan [2]. Its dried rhizomes are valued for their bitter taste, cool nature, and affinity to the liver meridian, exhibiting efficacy in clearing heat, detoxifying, reducing swelling, alleviating pain, and calming convulsions [3,4]. Clinically, it has been applied to treat conditions ranging from abscesses and snake bites to traumatic injuries and febrile seizures, often in combination with other herbs like *Lonicera japonica* and *Hedyotis diffusato* enhance therapeutic outcomes [5].

The medicinal properties of *Paris polyphylla* are largely attributed to its rich profile of steroidal saponins, which constitute over 80% of its identified compounds [6]. Among these, polyphyllin I (PPI) and polyphyllin II (PPII) serve as critical quality markers due to their pronounced pharmacological activities, including antitumor, hemostatic, analgesic, anti-inflammatory, and hepatoprotective effects [7,8]. However, the sustainable utilization of *Paris polyphylla* is challenged by its status as an endangered species, exacerbated by overharvesting and habitat loss. Moreover, the variability in saponin content influenced by drying methods and extraction techniques underscores the need for efficient and standardized approaches to ensure consistent quality and efficacy.

Conventional extraction methods for plant saponins, such as heat reflux and ultrasound-assisted extraction, often suffer from limitations like prolonged duration, high energy consumption, and suboptimal yield [9,10]. While emerging techniques like deep eutectic solvents (DES) have gained attention as green alternatives due to their low cost, biodegradability, and tunable properties, their application to *Paris polyphylla* remains underexplored [11,12]. Recent studies by Tian et al. [11] demonstrated that choline chloride-based DES systems achieve superior extraction efficiency for steroidal saponins compared to conventional solvents, while Vo et al. [12] highlighted the importance of hydrogen-bonding interactions between DES components and target compounds in enhancing solubility and mass transfer. The integration of ultrasonic assistance with DES (e.g., choline chloride–ethanol systems) leverages cavitation effects to enhance cell disruption and solute release, offering a promising avenue for improving extraction efficiency. Specifically, the synergistic combination of DES’s tunable solvation properties and ultrasound’s mechanical disruption creates an optimal environment for penetrating the rigid cell walls of *Paris polyphylla* rhizomes, particularly facilitating the release of high-molecular-weight steroidal saponins like polyphyllin I and II. Nevertheless, optimizing such processes requires systematic parameter evaluation, where orthogonal experimental designs can identify key factors like material-to-liquid ratio, extraction time, temperature, and ultrasonic power.

Accurate quantification of saponins is equally critical for quality control [13,14]. Although high-performance liquid chromatography (HPLC) has been widely used, it often lacks the sensitivity and speed needed for complex matrices [15,16]. Techniques like near-infrared spectroscopy (NIR) and ultraviolet spectroscopy (UV) face challenges in specificity and solvent consumption [17,18,19]. Advances in ultra-high-performance liquid chromatography coupled with tandem mass spectrometry (UHPLC-MS/MS) provide superior resolution, sensitivity, and structural elucidation capabilities, enabling precise detection of trace compounds such as PPI and PPII. For instance, the UHPLC-MS method established by Yang et al. successfully achieved the quantitative analysis of nine species of ligustrazine components in plasma samples, providing a reliable means for in vivo pharmacokinetic studies [20].

In this study, we address these gaps by developing an integrated approach combining DES-based extraction with UHPLC-MS/MS analysis for *Paris polyphylla*. Through orthogonal experimental design, we optimize key parameters to maximize yield while establishing a robust analytical protocol. Our work not only demonstrates the applicability of DES-intermittent pulsed ultrasound for efficient saponin recovery but also highlights the quantitative variations among seven *Paris polyphylla* varieties, providing a foundation for standardized quality evaluation and sustainable resource utilization. This research aligns with global trends in green chemistry and precision medicine, offering insights into the modernization of TCM through innovative technological integration.

## 2. Results and Discussions

### 2.1. Optimization of Extraction Process

**Figure 1:**
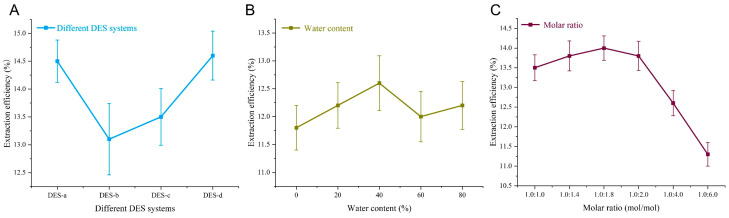
The varieties of the DESs: (**A**) Different DES systems, (**B**) water content (%), and (**C**) molar ratio (mol/mol) of DES-a. Data are presented as mean ± SD (*n* = 3).

We first investigate the extraction efficiencies for varieties of the DESs [11]. Figure 1A systematically evaluates the impact of different DES systems on the extraction efficiency of *Paris polyphylla saponins*. The screening of five DES systems (DES-a to DES-d; Table 1 revealed extraction efficiencies ranging from 13.1% to 14.6%, with the choline chloride–ethanol system (DES-a) achieving the highest efficiency of 14.5% and a deviation within 0.65%, indicating its superior stability and compatibility with target saponins. This superior performance can be attributed to specific structure-matrix interactions. In the DES-a system, chloride ions from choline chloride form strong hydrogen bonds with the hydroxyl groups on the sugar moieties of polyphyllins, enhancing their solubility. Simultaneously, the ethanol component provides a compatible hydrophobic microenvironment for the steroid aglycone. This synergistic interaction, along with DES-a’s optimal polarity for plant-cell penetration, establishes a molecular-level foundation for its high extraction efficiency. Additionally, three low-polarity systems, (A) choline chloride/n-hexane, (B) n-hexane alone, and (C) choline chloride/isopropanol, were also compared under identical extraction conditions (Figure S1). All three systems exhibited markedly lower extraction efficiencies than DES-a, confirming that merely matching low solvent polarity is insufficient for effective saponin recovery. These results underscore that DES-a’s balanced polarity and synergistic hydrogen-bonding network are critical for its superior performance in extracting *Paris polyphylla saponins*.

Further optimization of DES-a examined the critical role of water content (Figure 1B). As moisture increased from 0% to 80%, extraction efficiency initially rose, peaking at approximately 12.2% with a deviation within 0.5%, beyond which further hydration led to a gradual decline, suggesting that moderate water content enhances fluidity and solute accessibility while excessive water disrupts the eutectic network. Investigation of the molar ratio of choline chloride to ethanol demonstrated that a ratio of 1.0:1.8 yielded optimal performance with deviations constrained within 0.4%, underscoring the importance of balanced component stoichiometry in maintaining the structural integrity of DES and maximizing extraction efficiency (Figure 1C). These findings collectively establish DES-a with ~20% water content and a 1:1.8 molar ratio as an efficient and sustainable solvent system for saponin recovery. The formation of a true deep eutectic solvent (DES) was further confirmed by FTIR spectroscopy (Figure S2). A critical indicator was the significant broadening and redshift of the O-H stretching vibration (around 3400 cm^−1^) in the choline chloride–ethanol mixture compared to pure ethanol. This provides direct evidence of the breakdown of the initial hydrogen-bonding network in ethanol and the formation of new, stronger hydrogen bonds between the chloride ion (Cl^−^, HBA) and the hydroxyl group of ethanol (HBD). Shifts in the characteristic C-O and C-N stretching bands further corroborated this molecular interaction. These spectral features collectively verify that the mixture is not a simple solution but a DES with an extensive hydrogen-bonding network, which underpins its depressed melting point and enhanced extraction capability for steroidal saponins.

**Figure 2:**
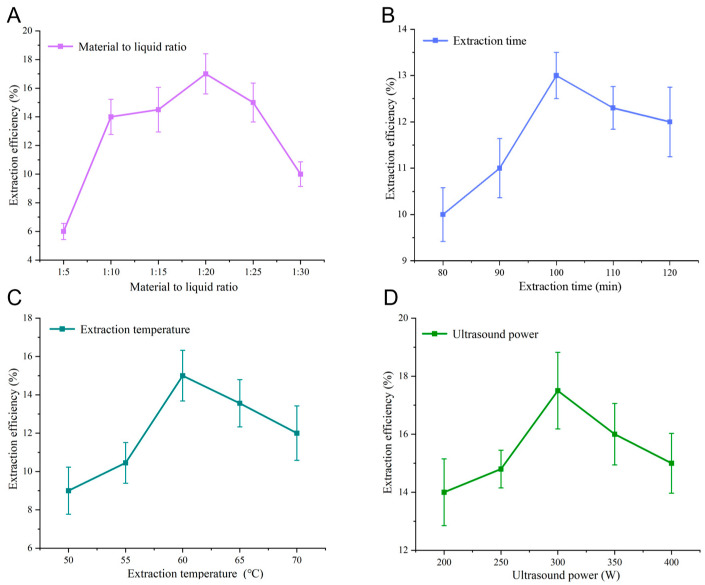
Effects of key extraction parameters on the saponin extraction efficiency using a choline chloride–ethanol system (DES-a): (**A**) material-to-liquid ratio (g mL^−1^), (**B**) extraction time (min), (**C**) extraction temperature (°C), and (**D**) ultrasound power (W). Data are presented as mean ± SD (*n* = 3).

Next, Figure 2 systematically illustrates the influence of four key extraction parameters on the yield of *Paris polyphylla saponins* using a deep eutectic solvent composed of choline chloride and ethanol (1.0:1.8 molar ratio). The single-factor experiments reveal that the material-to-liquid ratio significantly affects extraction efficiency, with an optimum value of 1:20 g·mL^−1^, beyond which dilution or saturation effects reduce saponin recovery (Figure 2A). Extraction time was optimized at 100 min, indicating that sufficient duration is necessary for mass transfer equilibrium, while prolonged exposure does not enhance yields (Figure 2B). Temperature exerted a pronounced impact, with 60 °C delivering maximal efficiency, likely due to improved solubility and diffusivity without triggering thermal degradation (Figure 2C). Ultrasound power optimization peaked at 300 W, where cavitation effects optimally disrupt plant cell walls to facilitate saponin release; higher power levels did not yield further improvement, suggesting possible saturation of acoustic energy utilization (Figure 2D).

Collectively, these results establish a refined set of operational conditions that balance efficiency and practicality, providing a robust foundation for subsequent orthogonal experimental designs and reinforcing the utility of DES-based extraction in natural product isolation.

Based on the single-factor experimental data, an L_9_(3^4^) orthogonal experimental design was employed to systematically optimize the extraction process of total saponins from *Paris polyphylla*, with the total extraction yield as the evaluation metric. Ultrasonic power was identified as the most influential factor affecting saponin yield, as evidenced by the range (R) values from the orthogonal test (Table 2), which decreased in the order: ultrasonic power (R = 3.15) > material-to-liquid ratio (R = 1.25) > extraction time (R = 1.06) > temperature (R = 0.62). Subsequent analysis of variance (ANOVA, Table 3) corroborates these findings, demonstrating that ultrasonic power is a highly significant factor (*p* < 0.01), while material-to-liquid ratio and extraction time are statistically significant (*p* < 0.05). In contrast, temperature shows no significant effect (*p* > 0.05), a finding that suggests the feasibility of lower extraction temperatures for developing energy-efficient and sustainable extraction protocols. The optimal extraction conditions were determined as A_2_B_3_C_1_D_1_, corresponding to a material-to-liquid ratio of 1:20 g·mL^−1^, an extraction time of 100 min, a temperature of 60 °C, and an ultrasonic power of 300 W. These results not only establish a robust and efficient extraction strategy but also highlight the critical role of ultrasonic power in enhancing the release of saponins, providing a mechanistic insight into process intensification for bioactive compound extraction from plant matrices.

Under the optimized extraction conditions established through orthogonal experimental design, validation experiments conducted in triplicate demonstrated the robust performance and broad applicability of the DES-intermittent pulsed ultrasound coupling method across seven distinct medicinal herbs from the Parisgenus and related species. As summarized in Table 4, the total extraction efficiency of saponins varied significantly among the samples, ranging from 2.04% for *Typhonium giganteum* to a markedly high yield of 16.51% for the root of *Paris polyphylla*, a variation likely attributable to intrinsic differences in saponin content and matrix structure among the plant materials. Crucially, the deviation values for the extraction efficiency were consistently low (0.32–4.04), underscoring the high reproducibility and reliability of the optimized protocol. The minimal variability observed between replicates, even across botanically diverse samples, confirms that the method is not only efficient but also exceptionally robust, effectively minimizing operational inconsistencies. This successful validation underscores the potential of this tailored extraction strategy for the standardized and quality-conscious analysis of bioactive saponins in a wide spectrum of medicinal plant resources.

### 2.2. Optimization of Chromatography and Mass Spectrometry Parameters

**Figure 3:**
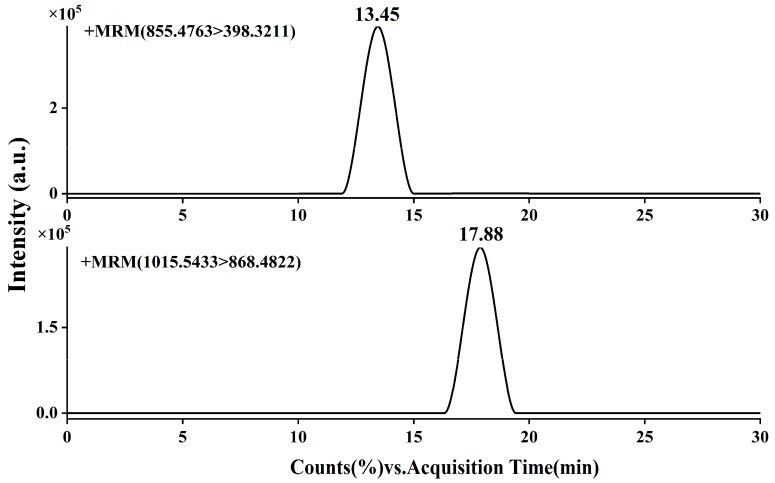
UHPLC chromatograms of polyphyllin I and polyphyllin II standards under optimized conditions.

Figure 3 illustrates the chromatographic separation of the two target saponins (PPI and PPII) under the optimized UHPLC conditions. Both analytes were well-resolved, with PPI eluting at 13.45 min and PPII at 17.88 min, demonstrating excellent peak shape and baseline separation free of significant matrix interference. This high-resolution separation provided a solid foundation for subsequent mass spectrometric detection.

**Figure 4:**
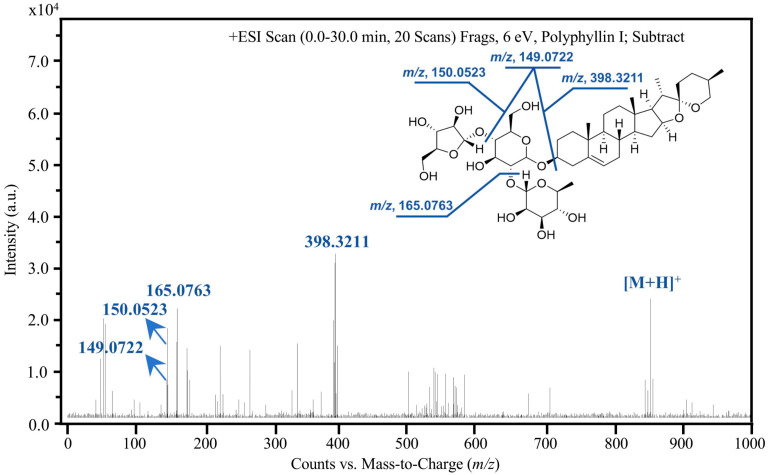
ESI-MS/MS product ion spectrum of PPI ([M+H]^+^ *m*/*z* 855.4763) in positive ion mode, highlighting the characteristic fragment at *m*/*z* 398.3211 ([C_27_H_42_O_2_]^+^).

**Figure 5:**
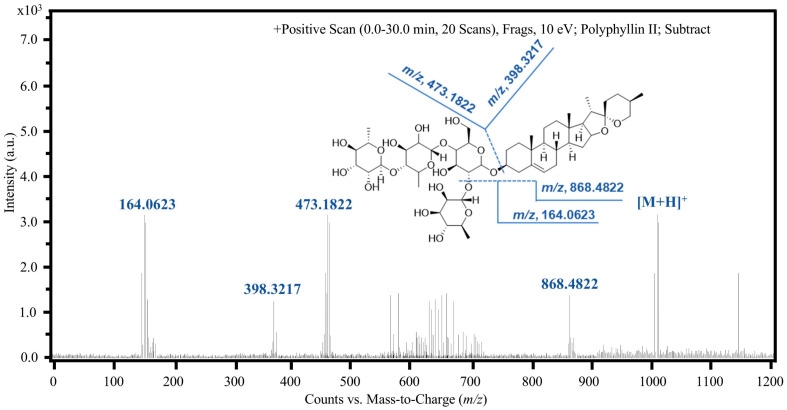
ESI-MS/MS product ion spectrum of PPII ([M+H]^+^ *m*/*z* 1015.5433) in positive ion mode, featuring the dominant fragment at *m*/*z* 473.1822 ([C_18_H_32_O_14_+H]^+^).

Further structural characterization was achieved through ESI-MS/MS analysis in positive ion mode, which yielded significantly higher response signals compared to the negative mode. As depicted in the product ion spectra (Figure 4 and Figure 5), the precursor ions [M+H]^+^ at *m*/*z* 855.4763 for PPI and *m*/*z* 1015.5433 for PPII were selectively fragmented to generate characteristic product ions. The most abundant fragments, *m*/*z* 398.3211 for PPI and *m*/*z* 473.1822 for PPII, were selected for multiple reaction monitoring (MRM). These correspond to the [C_27_H_42_O_2_]^+^ and [C_18_H_32_O_14_+H]^+^ moieties, respectively. This rigorous optimization of chromatographic and mass spectrometric parameters ensures high specificity and sensitivity, forming a robust analytical framework for the accurate quantification of these bioactive saponins in complex plant extracts.

### 2.3. Investigation of Methodological Verification Indicators

The method validation demonstrates exceptional analytical performance for the quantification of PPI and PPII. As illustrated in Figure S3, the UHPLC-MS/MS method exhibits a wide linear dynamic range (0.5–4000.0 ng mL^−1^ for PPI and 0.2–3000.0 ng mL^−1^ for PPII) with correlation coefficients (R^2^) exceeding 0.9976, indicating a robust linear relationship between concentration and detector response. The corresponding regression equations, detailed in Table 5, further confirm the method’s precision, with PPII showing a slightly wider linear range at the lower end. Notably, the method achieves impressive sensitivity, as reflected by detection limits of 0.4391 ng mL^−1^ for PPI and 0.1874 ng mL^−1^ for PPII, and similarly low quantitation limits. These results not only validate the reliability of the analytical procedure but also underscore its capability to detect trace levels of these bioactive saponins, making it well-suited for the precise quantification of complex plant extracts.

### 2.4. Analysis of Actual Samples

**Figure 6:**
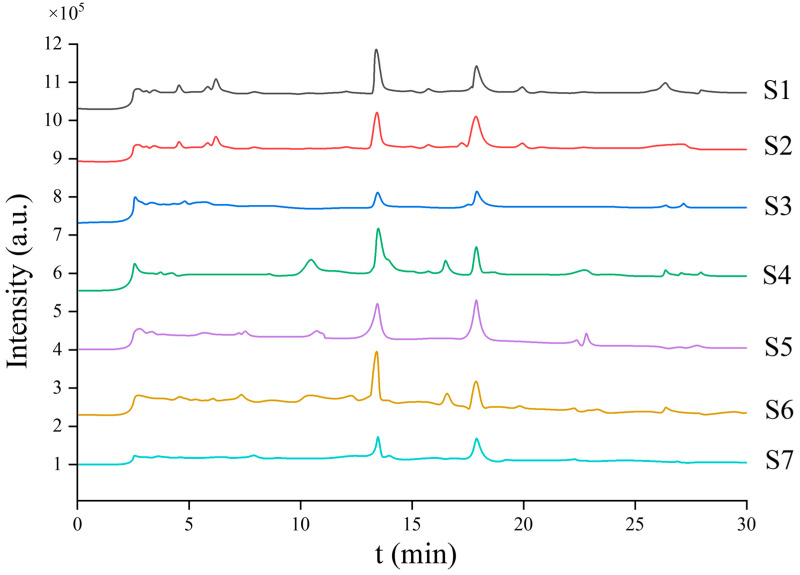
Stacked UHPLC-MS/MS chromatograms demonstrating the quantitative variation of PPI (~13.5 min) and PPII (~17.9 min) across seven *Paris polyphylla* samples (S1–S7).

Figure 6 clearly illustrates the successful application of the optimized UHPLC-MS/MS method for the simultaneous quantification of PPI and PPII across seven distinct medicinal plant samples (S1–S7). The chromatograms reveal well-resolved peaks for both analytes in all samples, with notable variations in signal intensity directly reflecting the divergent saponin content among the different plant species and tissues. These visual observations are robustly quantified in Table 6, which details substantial variations in PPI and PPII concentrations, with values ranging from 3.282 to 21.452 mg·g^−1^ and from 4.201 to 17.975 mg·g^−1^, respectively. This variability underscores the method’s critical utility for chemotaxonomic differentiation and quality assessment of medicinal materials. More importantly, the method’s exceptional accuracy and precision in complex plant matrices are unequivocally demonstrated by the satisfactory spike recovery rates, which span from 88.29% to 99.18% for PPI and 83.10% to 99.10% for PPII. The consistently low intra-day and inter-day relative standard deviations (RSD) for most samples confirm the high precision of the procedure. Slightly higher RSDs (4–5%) observed for S6 and S7 are attributed to the inherent heterogeneity of rhizome tissues and were further minimized through optimized grinding and moisture control, with all values remaining within accepted limits for plant matrix analyses.

## 3. Materials and Methodology

### 3.1. Materials and Instruments

The primary instruments used in this study included: an ultra-high-performance liquid chromatography-high-resolution mass spectrometer (UHPLC-HRMS, TSQ Orbitrap, Thermo Fisher Scientific, Waltham, MA, USA), a chromatographic column (Waters Acquity UHPLC BEH C18 column, 100 mm × 2.1 mm, 1.7 μm, Waters Corporation, Milford, MA, USA), an electronic analytical balance (Model Mark 120 A, Bohlamo Berlin Precision Instruments Co., Ltd., Berlin, Germany), a digital ultrasonic cleaner (Model KQ 2200 DA, Kunshan Ultrasonic Instrument Co., Ltd., Kunshan, China), and a high-speed refrigerated centrifuge (HITACHI, Hitachi Ltd., Tokyo, Japan).

The seven traditional Chinese medicinal materials used in the experiments were all purchased from the “Digital Yunyao” platform (https://www.shuziyunyao.com): *Paris polyphylla* Smith, *Typhonium giganteum*, *Iphigenia indica*, Chinese *Paris Rhizome*, Yunnan *Paris Rhizome*, Large-leaved *Paris Rhizome*, and Root of *Paris polyphylla*. Reference standards for PPI and PPII were obtained from Dalian Meilun Biotechnology Co., Ltd., Dalian, China (https://www.meilune.com). Chemical reagents such as ethanol and choline chloride were of analytical grade, while formic acid and acetonitrile were of chromatographic grade. All reagents were used directly without further purification.

### 3.2. Basic Information of PPI and PPII

**Figure 7:**
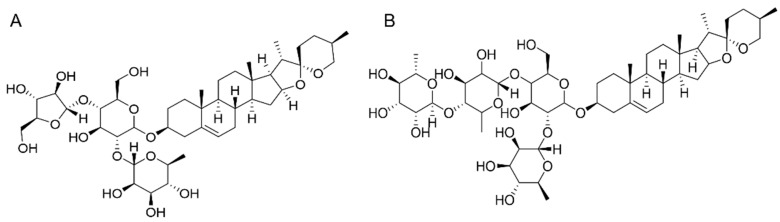
Chemical structural formulas of (**A**) PPI and (**B**) PPII.

Figure 7 displays the chemical structural formulas of PPI and PPII. Purities of PPI and PPII were 98. Based on prior stability studies [21], both compounds showed no significant degradation when maintained at temperatures up to 80 °C, ensuring their integrity under the employed extraction conditions (60–64 °C).

### 3.3. Ultrasonic-Assisted Preparation of Prepared Deep Eutectic Solvent Systems

In this study, four different solvent pairs were evaluated. For each DES, choline chloride was first dissolved in 8.0 mL of deionized water. This aqueous ChCl solution was then mixed with the respective hydrogen bond donor (e.g., ethanol for DES-a) according to a molar ratio of 1:1.8 for ChCl to HBD, as optimized from literature [22]. The mixtures were subjected to ultrasonic treatment in a 60 °C water bath, followed by magnetic stirring to ensure homogeneous DES formation. The final volume of each prepared DES was 20 mL. The specific compositions are detailed in Table 1.

### 3.4. Extraction Methods for Paris polyphylla Medicinal Material

The purchased seven types of *Paris polyphylla* medicinal materials were separately pulverized using a grinder and sieved through a 10-mesh sieve. Exactly 1.0 g of each medicinal powder was weighed and placed into a 200 mL round-bottom flask, to which a pre-prepared deep eutectic solvent system (DES-a) was added. Ultrasonic extraction was performed in a 60 °C water bath for 60 min, followed by additional stirring for 30 min using a constant-temperature magnetic stirrer to promote sufficient release of the active components.

After extraction, an appropriate amount of deionized water was used to transfer the reaction mixture to a centrifuge tube, which was then centrifuged at high speed in a refrigerated centrifuge (12,000 rpm, 10 min, 20 °C) to collect the supernatant. The supernatant was subsequently filtered through a 0.22 μm microporous membrane. The filtrate was concentrated to dryness using a rotary evaporator, and the residue was redissolved in 1 mL of methanol. The resulting solution was transferred to an injection vial for UHPLC-MS/MS analysis.

### 3.5. Optimization of Extraction Process Using Orthogonal Experimental Design

Building upon the preliminary single-factor experiments, this study employed an orthogonal experimental design to further optimize the extraction process for *Paris polyphylla*. Four key factors were selected for investigation: material-to-liquid ratio, extraction time, extraction temperature, and ultrasonic power. Each factor was assigned three distinct levels. The total extraction yield of saponins from *Paris polyphylla* was chosen as the evaluation index. An L_9_(3^4^) orthogonal array was utilized to design the experimental scheme. By comparing the influence of different factor levels on the extraction efficiency, the optimal combination of process parameters was identified. The specific experimental arrangement and the corresponding extraction results are presented in Table 7. Four factors examined are: (A) material-to-liquid ratio (1:16–1:20 g·mL^−1^), (B) extraction time (96–100 min), (C) temperature (60–64 °C), and (D) ultrasound power (300–320 W).

### 3.6. Chromatographic Parameter Conditions

The specific parameter settings for UHPLC chromatographic analysis are detailed in Table 8. A gradient elution method was employed for the mobile phase, where phase A consisted of a 0.1% formic acid aqueous solution and phase B was acetonitrile containing formic acid. The chromatographic flow rate was set at 0.2 mL min^−1^, and the injection volume was 5 μL. Under these conditions, satisfactory separation of PPI and PPII was achieved, providing high resolution and detection sensitivity, making the method suitable for the subsequent quantitative analysis of samples. Standard curves were established by plotting the peak areas against the concentrations of the active constituents. The content of each individual component was then calculated based on the corresponding calibrated concentration. The extraction efficiency (η) is calculated as follows:

(1) $$\eta \;=\;\frac{C_{i}\;\times \;V\;\times \;F\;}{m\times 10^{6}}\times 100\%$$

where *C_i_* represents the measured concentration (mg mL^−1^), *V* denotes the volumetric volume (mL), *F* is the dilution factor, and *m* refers to the sample mass (g).

### 3.7. Mass Spectrometry Parameter Conditions

The specific parameter settings for mass spectrometric analysis are detailed in Table 9. Detection was performed using the multiple reaction monitoring (MRM) mode under electrospray ionization positive ion (ESI+) conditions. High-purity argon was used as the collision gas to achieve effective collision-induced dissociation, while high-purity nitrogen served as both the desolvation gas and cone gas to maintain an optimal ionization environment. The capillary voltage was set at 2.5 kV, the cone voltage at 3.00 V, and the ion source temperature was maintained at 125 °C to ensure stable ion source operation and high-sensitivity mass spectrometric signals. These collectively optimized parameters guaranteed efficient ionization and specific detection of the target compounds, providing a reliable foundation for subsequent quantitative analysis.

### 3.8. Preparation of Standard Solutions

Polyphyllin I and polyphyllin II reference standards (1.0 mg each) were accurately weighed and separately dissolved in methanol. The solutions were quantitatively transferred to 10 mL volumetric flasks, diluted to the mark with methanol, and mixed thoroughly to obtain stock solutions at a concentration of 100 mg·L^−1^. A series of working standard solutions was subsequently prepared by appropriate dilution of the stock solutions. The concentration ranges of the working standards were 0.2, 0.5, 1.0, 2.0, 5.0, 10.0, 50.0, 100.0, 250.0, 500.0, 1000.0, 2000.0, 4000.0, and 5000.0 ng mL^−1^ for PPI, and 0.1, 0.2, 0.5, 5.0, 10.0, 50.0, 100.0, 500.0, 1000.0, 2000.0, and 3000.0 ng mL^−1^ for PPII. All solutions were prepared in suitable containers and mixed thoroughly before use to ensure homogeneity and accuracy for subsequent quantitative analysis and calibration curve construction.

## 4. Conclusions

In summary, an efficient and sustainable methodology for the extraction and quantification of saponins from *Paris polyphylla* was established using a DES combined with UHPLC-MS/MS. Through orthogonal experimental design, key extraction conditions including material-to-liquid ratio (1:20 g·mL^−1^), extraction time (100 min), temperature (60 °C), and ultrasonic power (300 W) were systematically optimized, yielding high recovery and reproducibility across seven plant samples. The developed UHPLC-MS/MS method demonstrated excellent analytical performance, with wide linear ranges, low detection limits, and high accuracy, enabling precise quantification of polyphyllin I and II. Significant variations in saponin content were observed among different varieties, particularly with the root sample achieving the highest extraction efficiency of 16.51%, underscoring the utility of this approach for chemotaxonomic discrimination and quality control. The integration of DES with ultrasound enhances extraction efficiency while aligning with green chemistry principles, offering an eco-friendly alternative to conventional techniques. This study provides a reliable and standardized platform for quality assessment of medicinal herbs, facilitating further phytochemical and pharmacological research.

## Figures and Tables

**Table 1:** Composition and molar ratios of the prepared deep eutectic solvent systems.

No.	Abbreviation	Solvent 1 (ChCl)	Solvent 2 (HBD)	Molar Ratio ^1^
1	DES-a	Choline Chloride	Ethanol	1.0:1.8
2	DES-b	Choline Chloride	Butanediol	
3	DES-c	Choline Chloride	Ethylene Glycol	
4	DES-d	Choline Chloride	Methanol	

^1^ The molar ratio refers to ChCl:HBD. HBD: Hydrogen bond donor.

**Table 2:** Range analysis of the L_9_(3^4^) orthogonal array design for the extraction process optimization.

No.	A ^1^	B ^2^	C ^3^	D ^4^	Extraction Efficiency (%) ^5^
1	1	1	1	1	9.16 ± 0.32
2	1	2	2	2	6.15 ± 0.28
3	1	3	3	3	7.02 ± 0.26
4	2	1	2	3	8.85 ± 0.34
5	2	2	3	1	10.12 ± 0.42
6	2	3	1	2	11.26 ± 0.40
7	3	1	3	2	9.76 ± 0.32
8	3	2	1	3	7.85 ± 0.32
9	3	3	2	1	10.53 ± 0.47
K1	28.45	29.16	29.98	27.11	
K2	28.16	31.45	28.64	31.66	
K3	30.15	29.78	30.36	31.47	
R	1.25	1.06	0.62	3.15	

^1^ A: material-to-liquid ratio (1:16, 1:18, 1:20 g·mL^−1^ for levels 1, 2, 3); ^2^ B: extraction time (96, 98, 100 min for levels 1, 2, 3); ^3^ C: extraction temperature (60, 62, 64 °C for levels 1, 2, 3); ^4^ D: ultrasonic power (300, 310, 320 W for levels 1, 2, 3). The notation A2B3C1D1 represents the optimal combination: material-to-liquid ratio 1:20 g·mL^−1^ (A2), extraction time 100 min (B3), temperature 60 °C (C1), and ultrasonic power 300 W (D1); ^5^ Mean ± SD, *n* = 3.

**Table 3:** Analysis of variance (ANOVA) of the factors influencing extraction yield.

Source of Variation	Sum of Squares	F Value	Significance (*p*-Value)
Material-to-liquid ratio (g mL^−1^)	1.5783	0.9435	<0.05
Extraction time (min)	1.3112	0.8624	<0.05
Extraction temperature (°C)	0.4645	0.2645	
Ultrasound power (W)	3.0764	1.2305	<0.01

**Table 4:** Total extraction efficiency of neutral DES intermittent pulse ultrasound coupled extraction from seven traditional Chinese medicinal materials.

Traditional Chinese Medicine	Extraction Efficiency (%)	Deviation of Efficiency (%)
*Paris polyphylla* Smith	14.33	2.87
*Typhonium giganteum*	2.04	0.32
*Iphigenia indica*	6.98	1.02
Chinese *Paris Rhizome*	7.47	1.76
Yunnan *Paris Rhizome*	14.63	3.22
Large-leaved *Paris Rhizome*	4.92	0.89
Root of *Paris polyphylla*	16.51	4.04

**Table 5:** Key analytical parameters for the UHPLC-MS/MS quantification of polyphyllin I and II.

Analyte	Linear Range (ng mL^−1^)	R^2^	Regression Equation	LOD (ng mL^−1^)	LOQ (ng mL^−1^)
polyphyllin I	0.5~4000.0	0.99952	Y = 2478.5592 + 391.6256x	0.4391	0.4832
polyphyllin II	0.2~3000.0	0.99763	Y = −1445.2323 + 285.7164x	0.1874	0.2143

**Table 6:** Quantitative analysis results of actual samples.

Sample	Polyphyllin I					Polyphyllin II				
	Content (mg·g^−1^)	Avg. Recovery (%)	Precision (RSD, %)		Accuracy (%)	Content (mg·g^−1^)	Avg. Recovery (%)	Precision (RSD, %)		Accuracy (%)
			Intra-Day	Inter-Day				Intra-Day	Inter-Day	
S1	7.983	93.28	1.27	1.33	97.26	8.921	97.16	1.09	1.22	91.99
S2	3.282	98.12	0.47	0.33	97.36	4.201	92.17	0.78	0.66	93.28
S3	7.391	90.18	1.42	1.22	97.27	9.721	83.10	2.07	1.87	94.27
S4	14.298	88.29	3.91	3.78	91.03	11.298	90.17	1.09	1.21	92.38
S5	9.276	95.28	2.10	2.07	94.67	5.292	96.18	0.99	1.03	98.71
S6	21.452	99.18	5.01	4.78	95.27	17.975	94.78	4.78	4.97	91.28
S7	17.294	97.91	4.57	4.23	96.35	16.392	99.10	3.89	3.76	98.13

**Table 7:** Factors and levels for the orthogonal experimental design.

Level	A	B	C	D
	Material-to-Liquid Ratio (g mL^−1^)	Extraction Time (min)	Extraction Temperature (°C)	Ultrasound Power (W)
1	1:16	96	60	300
2	1:18	98	62	310
3	1:20	100	64	320

**Table 8:** Chromatographic parameters of the target analytes.

Analyte	Polyphyllin I	Polyphyllin II
Chromatographic column	Waters Acquity UHPLC BEH C_18_ column (Milford, MA, USA)	Waters Acquity UHPLC BEH C_18_ column (Milford, MA, USA)
Mobile phase	A: 0.1% Formic acid in H_2_O B: Formic acid in acetonitrile	A: 0.1% Formic acid in H_2_O B: Formic acid in acetonitrile
Flow rate (mL min^−1^)	0.20	0.20
Elution gradient	0.0–1.0 min, 20%B; 2.0–3.0 min, 20–50%B; 3.0–5.0 min, 50–95%B; 5.0–28.0 min, 95%B; 28.0–29.0 min, 95–40%B; 29.0–29.5 min, 40–5%B 29.5–30.0 min, 5–5%B	0.0–1.0 min, 10%B; 1.0–1.5 min, 10–40%B; 1.5–2.0 min, 40–80%B; 2.0–3.0 min, 80–90%B; 3.0–29.0 min, 90%B; 29.0–29.5 min, 90–5%B 29.5–30.0 min, 5%B
Injection volume (μL)	5	5
Column temperature (°C)	38	38
Detection Wavelength (nm)	203	203
Retention Time (min)	13.45	17.88

**Table 9:** Mass spectrometry parameter information of the target substance.

Analyte	Polyphyllin I	Polyphyllin II
Ionization Mode	Positive (ESI+)	Positive (ESI+)
Precursor ion (*m*/*z*)	[M+H]^+^, *m*/*z*, 855.4763, C_44_H_70_O_16_	[M+H]^+^, *m*/*z*, 1015.5433, C_51_H_82_O_20_
Product ions	[C_27_H_42_O_2_]^+^, *m*/*z*, 398.3211; [C_6_H_12_O_4_+H]^+^, *m*/*z*, 149.0722; [C_5_H_10_O_5_]^+^, *m*/*z*, 150.0523; [C_6_H_12_O_5_+H]^+^, *m*/*z*, 165.0763;	[M-C_6_H_10_O_4_]^+^, *m*/*z*, 868.4822; [C_6_H_11_O_5_+H]^+^, *m*/*z*, 164.0623; [C_18_H_32_O_14_+H]^+^, *m*/*z*, 473.1822; [C_27_H_42_O_2_]^+^, *m*/*z*, 398.3217;
Collision energy (eV)	6	10
Instrument	Waters ACQUITY UPLC system coupled with a quadrupole time-of-flight mass spectrometer (Waters Corporation, Milford, MA, USA)	Waters ACQUITY UPLC system coupled with a quadrupole time-of-flight mass spectrometer (Waters Corporation, Milford, MA, USA)

## Data Availability

The original contributions presented in this study are included in the article and Supplementary Materials. Further inquiries can be directed to the corresponding author.

## Supplementary Materials

The following supporting information can be downloaded at: https://www.mdpi.com/article/10.3390/molecules31030473/s1, Figure S1. Different extraction systems: (A) choline chloride/n-hexane, (B) n-hexane, and (C) choline chloride/isopropanol. Data are presented as mean ± SD (*n* = 3). Figure S2. FTIR-ATR spectra at T = 298 K for different DES systems. Figure S3. UHPLC-MS/MS calibration curves for PPI and PPII standards.

## References

[1] Zhang Y., Fan Y.C., Zhang Y.C., Li Q., Su Y.Y., Xu C.S., Yu H.L., Wang C., Zhang J., Liao Z.X. (2025). Antitumor activity and mechanistic study of steroidal saponins from the rhizomes of *Paris polyphylla* var. *yunnanensis*. Phytochemistry.

[2] Zhou J., Liao B., Miao J., Chen X. (2025). *Paris* spp. (*Liliaceae*): A review of its botany, ethnopharmacology, phytochemistry, pharmacological activities, and practical applications. Front. Pharmacol..

[3] Ye X., Yang T., Pu X., Hu H., Chen J., Tan C., Tan X., Li S., Liu Y. (2025). The genus *Paris*: A fascinating resource for medicinal and botanical studies. Hortic. Res..

[4] Sha A., Li Y. (2025). Preparation, structural characterization, bioactivities, and potential clinical applications of the polysaccharides from *Paris polyphylla*: A review. Front. Pharmacol..

[5] Ma Y., Liu D., Cheng H., Bussmann R.W., He H., Guo Z., Liu B. (2019). Ethnobotanical study of medicinal plants used by Miao people in Jijiezi, Yunnan, China. Ethnobot. Res. Appl..

[6] Rawat J.M., Pandey S., Rawat B., Rai N., Preeti P., Thakur A., Butola J.S., Bachheti R.K., Wang C. (2023). Traditional uses, active ingredients, and biological activities of *Paris polyphylla* Smith: A comprehensive review of an important Himalayan medicinal plant. J. Chem..

[7] Liu J., Mu Y., Qi K., Li J., Hu Y. (2025). Regulation of anti-tumour effects of *Paris polyphylla* saponins via ROS: Molecular mechanisms and therapeutic potentials. Front. Pharmacol..

[8] Lin H., Chen B., Wang H., Pan F. (2025). Anti-neurodegenerative potential of polyphyllin: Mechanisms involving inflammation and oxidative stress modulation. Pharmacol. Discov..

[9] Li X., Guo J., Xu Y., Li S., Li N., Liu Q. (2025). Effects of heat reflux extraction on the content, antioxidant, and immune activity of polyphenols and flavonoids from hempseed threshing residues. PLoS ONE.

[10] Wang H., Deng L., Huang G. (2025). Ultrasound-assisted extraction and value of active substances in Muxu. Ultrason. Sonochem..

[11] Tian X., Liu J., Jiang L., Kong W., Fu Y., Qin L., Cui Q. (2024). Efficient extraction and optimization procedures of polyphyllins from *Paris polyphylla* var. *chinensis* by deep eutectic solvent coupled with ultrasonic-assisted extraction. Microchem. J..

[12] Vo T.P., Ho T.A.T., Truong K.V., Ha N.M.H., Nguyen D.Q. (2024). Combining novel extraction techniques with natural deep eutectic solvent to acquire phenolic and terpenoid compounds from *Paris polyphylla* roots. J. Agric. Food Res..

[13] Xiao X., Yuan Z., Li G. (2014). Separation and purification of steroidal saponins from *Paris polyphylla* by microwave-assisted extraction coupled with countercurrent chromatography using evaporative light scattering detection. J. Sep. Sci..

[14] Wu Z., Zhang J., Xu F., Wang Y., Zhang J. (2017). Rapid and simple determination of polyphyllin I, II, VI, and VII in different harvest times of cultivated *Paris polyphylla* Smith var. *yunnanensis* (Franch.) Hand.-Mazz by UPLC-MS/MS and FT-IR. J. Nat. Med..

[15] Shi Y.X., Xu L., Wang X., Zhang K.K., Zhang C.Y., Liu H.Y., Ding P.P., Shi W., Liu Z.Y. (2024). *Paris polyphylla* ethanol extract and polyphyllin I ameliorate adenomyosis by inhibiting epithelial-mesenchymal transition. Phytomedicine.

[16] Thakur U., Shashni S., Thakur N., Rana S.K., Singh A. (2023). A review on *Paris polyphylla* Smith: A vulnerable medicinal plant species of a global significance. J. Appl. Res. Med. Aromat. Plants.

[17] Han M., Wang Y. (2025). Mining the potential quality marker and predicting the total flavonoid content of *Paris polyphylla* var. *yunnanensis* based on information fusion. Microchem. J..

[18] Pei Y., Zuo Z., Zhang Q., Wang Y. (2019). Data fusion of Fourier transform mid-infrared (MIR) and near-infrared (NIR) spectroscopies to identify geographical origin of wild *Paris polyphylla* var. *yunnanensis*. Molecules.

[19] Zhong C., Li L., Wang Y. (2025). Comparative analysis of flavonoids in *Paris polyphylla* var. *yunnanensis* under different climatic zones using FT-NIR spectroscopy, UPLC-ESI-MS/MS, and chemometrics. J. Appl. Res. Med. Aromat. Plants.

[20] Yang Y., Zhang J., Jin H., Zhang J., Wang Y. (2016). Quantitative analysis in combination with fingerprint technology and chemometric analysis applied for evaluating six species of wild *Paris* using UHPLC-UV-MS. J. Anal. Methods Chem..

[21] Li Y., Wen R., Yang W., Xu H., Xie Q., Wang L., Sun H., Zhang H., Xia C. (2024). Multimodal integrated strategy for the discovery and identification of antiplatelet aggregation Q-markers in *Paris polyphylla* var. *yunnanensis*. Biomed. Chromatogr..

[22] Delgado-Mellado N., Larriba M., Navarro P., Rigual V., Ayuso M., García J., Rodríguez F. (2018). Thermal stability of choline chloride deep eutectic solvents by TGA/FTIR-ATR analysis. J. Mol. Liq..

