# Supplementary material for: Composition Study of Polyphyllin in Paris polyphylla by Ultrasound-Assisted Deep Eutectic Solvent Extraction Combined with UHPLC-MS/MS
Source: Molecules. 2026 Jan 29;31(3):473. doi: 10.3390/molecules31030473 (PMC12899768; doi:10.3390/molecules31030473)
Supplement: Supplementary file 1 [file molecules-31-00473-s001.zip › molecules-4073105-supplementary.pdf]

Supplementary Material

Composition Study of Polyphyllin in *Paris polyphylla* by Ultrasound-Assisted Deep Eutectic Solvent Extraction Combined with UHPLC-MS/MS

Jinyu Guo, Jiajia Liu, Minlong Li, Zhenlin Tan, Huayin Lu and Yuting Zhou*

School of Pharmacy, Youjiang Medical University for Nationalities, Baise 533000, China.

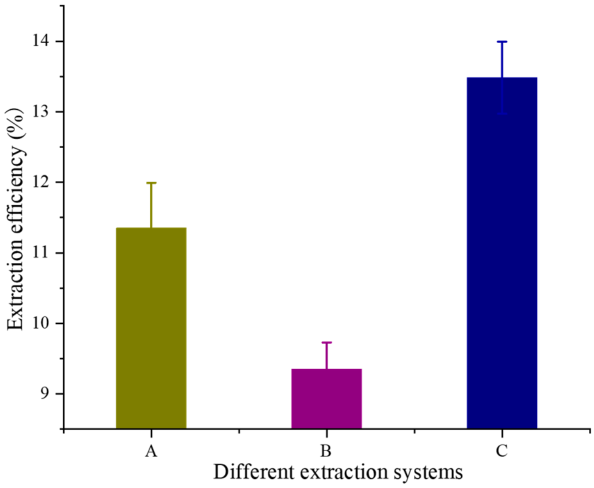

Figure S1. Different extraction systems: (A) choline chloride/n-hexane, (B) n-hexane, and (C) choline chloride/isopropanol. Data are presented as mean ± SD (n = 3).

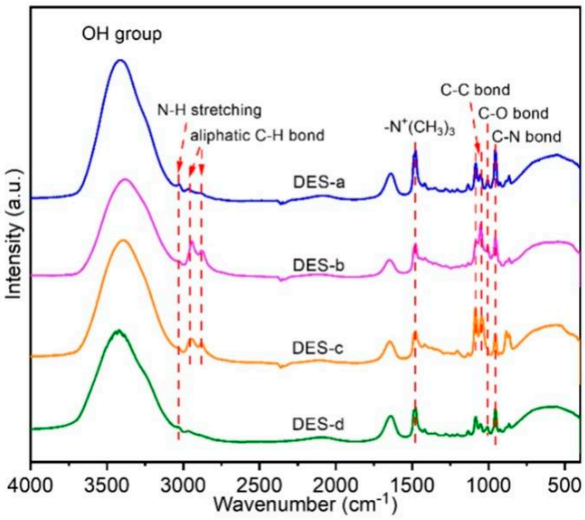

Figure S2. FTIR-ATR spectra at T = 298 K for different DES systems.

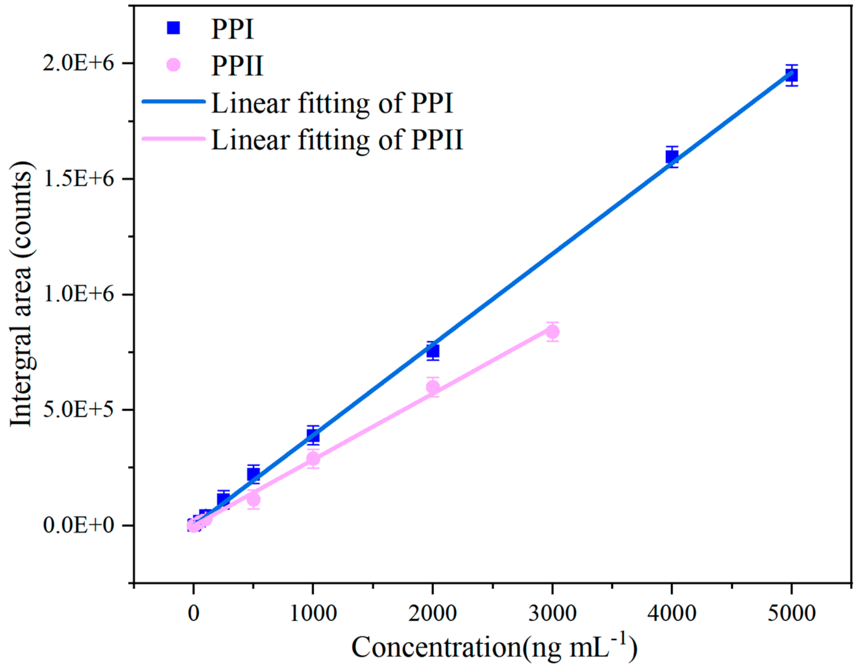

Figure S3. UHPLC-MS/MS calibration curves for PPI and PPII standards.
